# Anise (*Pimpinella anisum*) and fennel (*Foeniculum vulgare*) essential oils and their combination as natural and safe housefly (*Musca domestica*) ovicides

**DOI:** 10.3389/finsc.2026.1817242

**Published:** 2026-04-23

**Authors:** Hataichanok Passara, Chamroon Laosinwattana, Tanapoom Moungthipmalai, Kouhei Murata, Mayura Soonwera

**Affiliations:** 1 Office of Administrative Interdisciplinary Program on Agricultural Technology, School of Agricultural Technology, King Mongkut’s Institute of Technology, Bangkok, Thailand; 2 School of Agriculture, Tokai University, Kumamoto, Japan

**Keywords:** *Eisinia fetida*, essential oils, *Foeniculum vulgare*, housefly eggs, *Pimpinella anisum*, synergistic ovicides

## Abstract

Essential oils (EOs) are potential natural substitutes for synthetic insecticides. This study investigated the ovicidal efficacy and environmental safety of single and combined formulations of anise (*Pimpinella anisum*) and fennel (*Foeniculum vulgare*) EOs against the housefly, *Musca domestica*. For efficacy evaluation, all formulations were stored at 25 °C and their ovicidal activity and stability were assessed after one year, using *α*-cypermethrin as a standard insecticide control. A 1:1 anise–fennel EO mixture showed the highest activity, achieving complete ovicidal effect (100%) for up to 6 months under laboratory conditions, with LC_50_ values of 0.07–0.08 µL egg⁻¹. After one year of storage, the formulation remained highly effective, with 95% inhibition and an LC_50_ of 0.08 µL egg⁻¹. The combined formulation exhibited strong ovicidal synergy and was more effective than individual EO formulations and α-cypermethrin at all time points (synergistic inhibition index: 12.9–16.3). Morphological observations of treated eggs revealed damage to the hatching line, aeropyles, and plastron. In a separate safety assessment, toxicity was evaluated using the non-target earthworm *Eisenia fetida*, where all EO treatments showed no apparent toxicity, while α-cypermethrin was highly toxic. These results demonstrate that the 1:1 combined anise and fennel EO formulation is an effective, stable, and environmentally safe ovicidal agent for sustainable housefly control.

## Graphical Abstract

**Figure:**
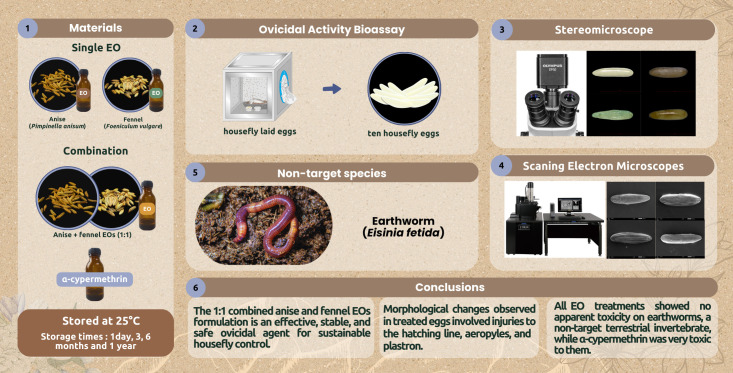

## 1 Introduction

Houseflies are mechanical vectors for a variety of pathogens that cause serious diseases in human and livestock such as diarrhea, dysentery, shigellosis, food-borne diseases, coronavirus infection, anthrax disease, lumpy skin disease (LSD) and Newcastle disease (1). Preventing humans and animals from getting bitten and contract a disease by houseflies is still one of the greatest challenges, as numerous control tactics are based on synthetic insecticides for their management (2). It is difficult to monitor and control housefly populations, and it usually leads to significant financial losses, especially since these flies are resistant to numerous commonly used chemical insecticides (3). The widespread and extended use of synthetic insecticides have consistently shown hazardous effects such as human health risks, environmental pollution, and non-target population suppression. Non-target creatures such as soil invertebrates and aquatic organisms (4). Furthermore, chronic exposure to chemical insecticides has been related with severe human health maladies including several types of cancer, Alzheimer’s diseases, Parkinson’s diseases, asthma as well as allergic reactions (5). Additionally, synthetic insecticides remain in soil and water residues, pollute the air, enter the food chain, disrupt nitrogen fixation and biodiversity and inflict serious damages on bird populations as well as marine ecosystems (6). In this context, novel organic insecticides (termed botanicals) from natural plant sources including EOs and other bioactive compounds are lower risks for human health and environmental pollution as well as safer alternatives compared to modern chemical insecticides (6, 7). Botanical essential oils have become good tools for managing insect pests, including housefly populations (8). These products are rich in diverse bioactive ingredients that act as repellents or toxic substances against houseflies in every stage of their development. Moreover, essential oils are environmentally safe for humans and non-target organisms. They decompose quickly, leave less residue, and are lower risk against nontarget species. They are some of the most promising alternatives for sustainable management of houseflies (9).

Currently, more and more entomological research studies have focused on botanical insecticides for the control of insect vectors such as houseflies with special reference to their larvicidal, pupicidal, and adult repellent activity, but few studies have focused on ovicidal activity (10). In the few ovicidal studies that have been published, the plant species with the potential to be used as ovicides against housefly populations are notable for disrupting and injuring their eggs at actual breeding sites so that emerging generations can be affected. Great dependence on synthetic insecticides is reduced, toxic chemical residues in nature decreased, and environmentally compatible management tools are obtained (11). A number of essential oils (EOs) and their main bioactive components have shown strong ovicidal and oviposition deterrent activity against houseflies. These EOs are extracts lemongrass (*Cymbopogon citratus*) essential oil, star anise (*Illicium verum*) essential oil and peppermint (*Mentha piperita*), anise (*Pimpinella anisum*), carom seeds (*Trachyspermum ammi*), black cumin (*Centratherum anthelminticum*), dill (*Anethum graveolens*), fennel (*Foeniculum vulgare*) etc. (**Table 1**). Plant essential oils act ovicidally on the eggs of houseflies and other insects by interfering with the plastrons or hatching line, resulting in embryonic death (11). Essential oils of star anise, lemongrass, and their active components *trans*-anethole and geranial are potent housefly ovicides. They cause obstructed aeropyles and micropyles, followed by retarded embryogenesis (11). Also, a blend of eucalyptus (*Eucalyptus globulus*) and zedoary (*Zingiber zedoria*) essential oils in a 1:1 ratio was demonstrated to be ovicidal against the eggs of head louse (*Pediculus humanus* capitis), by blockade of aeropyles and destruction to embryonic respiratory system leading to an embryonic death (15). Notably, although plant essential oils are toxic to housefly, the oils are often regarded as being environmentally safe to a number of non-target organisms (such as pollinators, predators, and earthworms) (13, 16–20). Furthermore, essential oils are used as food additives, spices, and traditional medicine in many parts of the world: especially those obtained from plants of Apiaceae family (Umbelliferae) (14). Of these, anise and fennel oils have been shown to exhibit good insecticidal, oviposition deterrent and repellent activities against housefly and other vectors (10, 14). Crucially, these essential oils are environmentally safe and have little effects on non-target species (21).

**Table 1:** Reported ovicidal and oviposition deterrent activities of EOs and active constituents against housefly (*M*. *domestica*) eggs.

EOs/EO constituents	Concentrations	Deterrent (%)	Inhibition Rate (%)	Action	Ref.
Geranial	1.0%	–	53.2	Toxic to the nervous system and disturbed embryo development	(11)
*Trans-*anethole	1.0%	–	58.6	Inhibited the enzyme acetyl cholinesterase (AChE)	(11)
Lemongrass	1.0%	–	0.5	Inhibited the enzymes AChE and butyrylcholinesterase (BChE)	(11)
Star anise	1%	–	1.0	Reduced AChE activity	(11)
Lemongrass + *trans-*anethole	2%	–	94.5	Blocked aeropyles, micropyle and embryonic development	(11)
Star anise + geranial	2%	–	96.2	Blocked hatching line and plastron	(11)
Star anise	10%	100	97.3	Reduced oviposition potential and toxic to the nervous system	(12)
Peppermint	10%	88.6	22.7		(12)
Cassumunar ginger (*Zingiber cassumunar*)	10%	97.2	9.0		(12)
lavender (*Lavandula angustifolia*)	10%	88.1	5.3		(12)
Clove (*Syzygium aromaticum*)	1650 µg/cm^2^	100	–	Reduced oviposition potential	(13)
Ylang-ylang (*Cananga odorata*)	1650 µg/cm^2^	100	–		(13)
Citronella grass	1650 µg/cm^2^	98.9	–		(13)
Anise	10%	100	–	Reduced oviposition potential and toxic to the nervous system	(14)
Carom seeds	10%	100	–	Reduced oviposition potential	(14)
Black cumin	10%	100	–		(14)
Dill	10%	100	–		(14)
Fennel	10%	100	–	Reduced oviposition potential and toxic to the nervous system	(14)

Besides the target potency, the environmental safety aspect in terms of insecticides applied for housefly control to soil ecosystem needs to be addressed. Synthetic insecticides used in fly breeding places (animal manure, refuse dump, and organic waste) often find their way into the soil and persist there with potential negative effects on organisms living in the soil. Earthworms are one of the most sensitive non-target soil invertebrates to such pollutants. They contribute a critical role to ecological functions: promoting soil aeration, decomposing organic matters, cycling nutrients, and retaining soil structure. Several studies have shown that contact with chemical insecticides negatively influences survival, growth, reproduction, and burrowing behaviour of earthworms, in turn affecting soil health and ecosystem functioning (22–24). Earthworm is hence a well-known environmental biomonitor. It is a sentinel species and a bioindicator of the ecotoxicity of pesticides in the soil. Their intimate contact with soil, high uptake of soil particles and organic matter, and bioaccumulation potential for chemical residues render them a particularly good candidate for sensing early alterations in ecology due to toxic compounds (25, 26). It is known that changes in earthworm mortality and behaviour have been used as early warnings of environmental risk in terrestrial ecosystems, and standardized earthworm toxicity tests are internationally adopted for environmental risk assessment (22). Thus, testing earthworm sensitivity toward EO-based ovicides is ecologically valid and scientifically sound for assessing the non-target safety and environmental suitability of products.

Since storage period is a fundamental factor that affects stability and biological efficacy of EO-based ovicides (16) and since prolonged storage could induce the loss of bioactivity over time (20), this study investigated the performance of EO formulations systematically during storage for one year. The ovicidal activity of an EO formulation was determined at predetermined time points under standardized storage conditions in order to determine the temporal differences in killing insect eggs (27, 28). This experiment provided a possibility to assess the capacity and stability of ovicidal activity over long-term storage, thus obtaining information for practical shelf life of EO originating ovicides (16, 29). This knowledge is crucial for identifying the potential of EO formulations as sustained control agents in insect and vector control programs (28).

Therefore, this research aimed to assess the ovicidal efficacy and storage stability of two types of single EOs (anise and fennel) and their combined formulation on housefly eggs. These EOs were chosen on the basis of previously demonstrated insecticidal activity, oviposition deterrent effect, and safety to non-target organisms as well as their popularity in consumption in foods and traditional medicine (14, 19). Laboratory bioassays were performed to evaluate the ovicidal efficacy, including the possible synergistic effect of combined EOs, and stability of EO formulations during storage for 1 year. In addition, optical microscopy and SEM were used to observe morphological changes in housefly eggs after the EO treatments, which may clarify the relationship between the ovicidal action of EOs and their mode of action.

## 2 Materials and methods

### 2.1 Housefly breeding

Housefly eggs were originally obtained from the Entomological Laboratory, School of Agricultural Technology, King Mongkut’s Institute of Technology Ladkrabang (KMITL), Bangkok, Thailand. The colony was reared under environmentally controlled conditions (25.8 ± 1.8 °C, 70.5 ± 2.5% relative humidity, and L12: D12 h light: dark). The eggs hatched and developed to the first instar, second instar (reached by 24–48 h), third instar larvae, and pupae within 10 days when reared on the diet of fresh mackerel and wet pet food (1:1). The pupae were kept starved and after 72–120 h, they emerged as adult and separated into either male or female adults to be fed on a diet containing milk powder: multivitamin syrup: mineral water (1:1:8). After 48–72 h, gravid females oviposited on the same steamed mackerel pieces and also a wet pet food substrate (1:1). Newly laid eggs of 1–3 h old were collected for ovicidal bioassays in accordance with the method reported by Passara et al. (11).

### 2.2 Plant essential oils and chemicals

Essential oil of fennel (CAS No. 8024-24-6) was provided by Chemipan Corporation Company Limited, Khan Na Yao, Bangkok, Thailand, and anise essential oil (CAS No. 8007-70-3) was obtained from Sigma-Aldrich Co., Ltd., St. Louis MO, USA. The two EOs and their 1:1 mixture were prepared as 70% (v/v) stock solutions in ethyl alcohol. A 10% (w/v) emulsifiable concentrate (EC) of α-cypermethrin was used as a standard insecticide. Ethyl alcohol and α-cypermethrin were obtained from M&P Impex Corporation Co., Ltd., Ladkrabang, Bangkok, Thailand. *Trans*-anethole (76.6–97.9%) was the major constituent of anise EO, and estragole (13.0%), 1,8-cineole (4.7%), and linalool (1.5–7.4%) were minor components (30–32). Similarly, *trans*-anethole (65.1–76.2%) was the major constituent of fennel EO followed by fenchone (10.9–25.6%), estragole (9.5%), methyl chavicol (3.4%), α-pinene (1.5%) and limonene (1.3%), which were present in lower amounts. In both EOs, all other constituents were less than 1% of the total composition (33, 34).

### 2.3 Single EO and combined EO treatments

The treatment concentrations used in the present study were selected based on our previous studies (11, 20, 32). The two EOs were mixed in an equal ratio, and their effect was evaluated at 0.05; 0.1; 0.25; 0.5, and 1 µL/egg under controlled laboratory conditions (25,8 ± 1,8 °C/70,5 ± 2,5% RH). The EO formulations were filtered and transferred into 100 mL amber screw-capped bottle (brown bottle), then maintained under the laboratory conditions: 25 °C, 71% RH, and alternating 12 h light and dark periods. The ovicidal effect was determined at 1, 30, 90, 180 and 365 days of storage. Bioassays were performed to evaluate ovicidal activity, by submitting the eggs of housefly to the treatments. All treatments, positive control (α-cypermethrin), and negative control (distilled water) were conducted with the same experimental procedures.

### 2.4 Ovicidal activity bioassay

The ovicidal activity of single EO formulation and combined EO formulation was tested with a topical application method, as previously described by (11), in five replications. In each experiment, 1 g steamed mackerel piece and wet pet food (1:1) mixture were placed in a Petri dish (50 mm diameter), onto which ten fertile housefly eggs were carefully deposited. The defined amounts of the EO formulations (0.05, 0.1, 0.25, 0.5 and 1.0 µL per egg) were applied directly onto the eggs using a micropipette at each concentration and storage period tested. Concomitant treatments with α-cypermethrin and distilled water were used as the positive and negative controls, respectively. Egg mortality was recorded 48 h after treatment under an optical microscope. Eggs were scored as dead if the hatching line was unopened, or it had opened but there was a still born embryo (11).

The egg inhibition rate (IR) was calculated using Equation 1, as described by Passara et al. (11),

(1) IR%=(A/B)×100

where A is the total number of unhatched eggs, and B is the total number of treated eggs.

The decreasing inhibition index (DII) was calculated from Equation 2 (11):

(2) $$\text{DII}={\text{LC}}_{50},\text{ t}/{\text{LC}}_{50},1,\;$$

where LC_50_, t is the lethal concentration at which 50% of eggs were unhatched after 2 days after exposure to samples stored for 1 year, and LC_50_, 1 is the lethal concentration at which 50% of eggs were unhatched after 2 days after exposure to samples stored for 1 day.

The Effective Inhibition Index, EII treat, was calculated from Equation 3 (11, 20):

(3) $$\text{EII treat}={\text{LC}}_{50}\text{ treat}/{\text{LC}}_{50}\text{ cyper}$$

where LC_50_ treat is the lethal concentration at which 50% of eggs were unhatched by treatment, after 2 days vs α-cypermethrin.

EII indicates the relative effective ovicidal activity, with EII < 1 signifying that the treatment was more effective than α-cypermethrin, whereas EII > 1 indicates that the treatment was less effective.

The Synergistic Inhibition Index, SII, is the relative efficacy of the combined EO formulation over a single EO formulation at the same strength. SII was calculated using Equation 4 (11, 20):

(4) $$\text{SII}={\text{LC}}_{50}\text{ sing}/{\text{LC}}_{50}\text{ com}$$

where LC_50_ sing is the 50% lethal concentration of each single EO, and LC_50_ com is the 50% lethal concentration of the combined EO formulation. SII indicates relative synergy, with SII > 1 implying synergy, whereas SII < 1 signifies no synergy.

### 2.5 Ovicidal structural changes from microscopy

After ovicidal bioassays, the internal morphological aberrations of treated eggs and untreated eggs were observed under a scanning electron microscope (SEM). SEM images were taken at the Scientific and Technological Research Equipment Centre, Chulalongkorn University, Bangkok, Thailand as previously described (11, 20, 32, 35). Based on the SEM images, certain structural changes were observed in eggs treated with single and combined EO formulation, when compared to untreated groups. At 48 h after treatment, both treated and untreated eggs were post-fixed in 95% (v/v) ethanol for 90 min and then dehydrated with three changes of 100%-ethanol, each for 90 min. The samples were then dried in a CO_2_ critical point dryer. Dried eggs were mounted on aluminum studs with double sided adhesive tape and gold sputter-coated to a thickness that allowed for good image resolution. Photomicrographs of eggshell structures, i.e., hatching lines, plastrons, and aeropyles were taken with scanning electron microscope JSM-6610LV scanning electron microscope of JEOL Co. Ltd (Tokyo, Japan).Furthermore, photos of abnormal eggshell surface morphology and changed color of eggs as well as dead embryos were captured with a Nikon ^®^ C-PS microscope (Nikon Cell Innovation Co., Ltd., Tokyo, Japan) attached to a Nikon ^®^ DS-Fi2 digital camera at the Agricultural Microscopy Centre, King Mongkut’s Institute of Technology Ladkrabang (KMITL), Bangkok, Thailand (11).

### 2.6 Assessment of non-target safety using earthworm bioassay

Toxicity test with *E. fetida*: The toxicity test was performed in accordance with the OECD testing guidelines (25). Two thousand earthworms were obtained from the KMITL organic farm on 27 November 2025 (1,000 individuals per treatment). They were classified in a black plastic container (850 mm diameter × 250 mm height). Three concentrations of treatment (100, 200 and 400 µL/kg) were tested. The container was filled with test soil mass of 5 kg (a mixture of organic fertilizer, cow manure, coconut husk and organic soil at the ratio of 1:1:1:1), having a pH range of 6.5–7.0 and moisture content to form a consistency of 65% paddy field capacity (36). Experiments were carried out at 25 ± 2 °C with a 12 h light/12 h dark photoperiod. Each treatment was mixed by hand with 1 mL of respective treatment into 1 kg test soil, placed in a black plastic container (250 mm diameter × 200 mm height) where 10 earthworms were released. The uniformity of the biological responses was the evaluation criterion. Earthworm responses were regularly checked, and no spatial variability in response was observed, showing that the test substances were uniformly distributed. Each treatment included five replicates and was carried out simultaneously with the negative (distilled water) and positive controls (α-cypermethrin). The percentage mortality was observed after 1, 5, 10 and 15 days post-exposure.

The earthworm mortality rate (%M) was calculated using the following Equation 5 (36):

(5) Mortality rate (%M)=D/T

where D represents the number of dead earthworms, and T denotes the total number of treated earthworms.

### 2.7 Statistical analysis

All bioassays were carried out in a completely randomized design (CRD), and the data were analyzed using IBM SPSS Statistics software version 28 (IBM Corp., Armonk, NY, USA). Average egg inhibition rates and average mortality from non-target organism bioassays were tested for significant differences using one-way ANOVA, coupled with Tukey’s HSD test to make comparisons of treatment means (P < 0.05) (11, 37). Ovicidal effectiveness and hazards to earthworms were also estimated in terms of the concentrations that provoked 50% inhibition (LC_50_) of egg hatching of flies and 50% mortality (LC_50_) of adult earthworms after 48 h exposure, respectively, through probit analysis. Hatch inhibition or mortality was the response variable in these analyses. Treatment was a fixed factor and exposure time a covariate.

## 3 Results

### 3.1 Ovicidal activity

**Figure 1:**
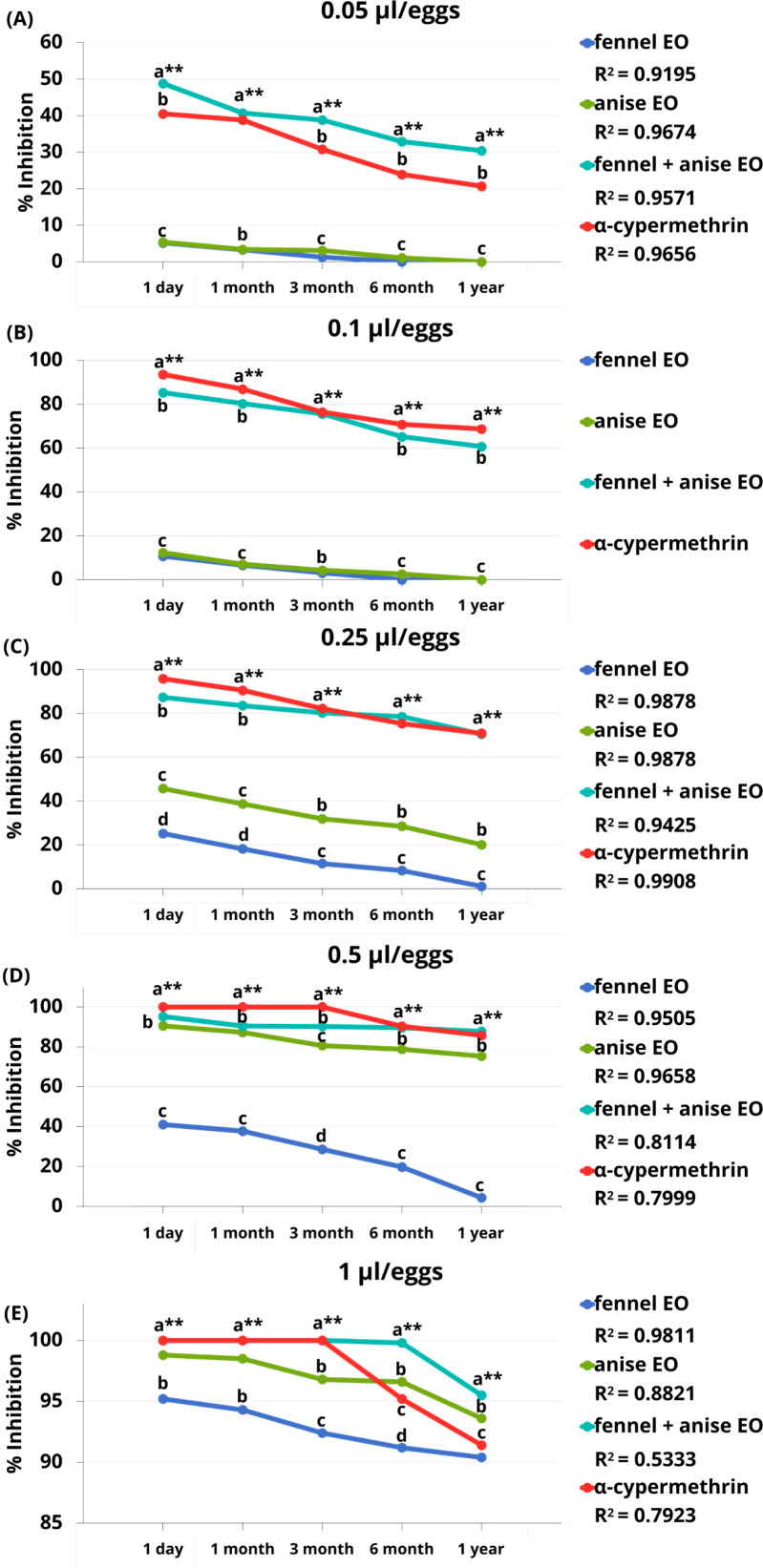
Inhibition rates versus storage time of treatments and α-cypermethrin against housefly eggs at 0.05 μL/eggs **(A)**, 0.1 μL/eggs **(B)**, 0.25 μL/eggs **(C)**, 0.5 μL/eggs **(D)**, 1 μL/eggs **(E)**. Values that are accompanied by different letters (a–d) show significant differences between the treatments. ** for p < 0.01, the coefficient of determination value (R^2^).

**Figure 1** illustrates the ovicidal activity, expressed as the egg inhibition fraction, of single and combined EO formulations against housefly eggs at doses of 0.05, 0.1, 0.25, 0.5, and 1.0 µL per egg over storage periods ranging from 1 day to 1 year. All treatments exhibited strong inhibitory effects on egg hatching at the 1-day storage interval; however, the inhibition rate gradually declined with increasing storage duration from 1 month to 1 year. In addition, the physical characteristics of the combined EO formulation, including color, clarity, and odor remained visually comparable to those of the freshly prepared formulation after 1 day of storage.

The results demonstrated that all treatments applied at the highest dose (1.0 µL per egg) achieved strong ovicidal activity, with egg inhibition rate exceeding 90%. At a lower dose of 0.5 µL per egg, the combined EOs formulation and cypermethrin exhibited comparably high levels of efficacy, whereas anise EO alone showed moderately reduced activity and fennel EO alone generally displayed approximately half of the inhibitory effect. At the lowest tested dose (0.05 µL per egg), the combined EO formulation exhibited significantly greater ovicidal activity than the individual EOs and achieved inhibition levels comparable to those of cypermethrin. Over extended storage periods of up to 6 months and 1 year, the EO combination consistently demonstrated slightly higher efficacy than cypermethrin. Although ovicidal activity declined with decreasing dose and prolonged storage time, the fennel–anise EO combination remained as effective as the synthetic insecticide cypermethrin. In contrast, the distilled water control resulted in a 100% egg hatching rate, corresponding to 0% inhibition.

**Figure 2:**
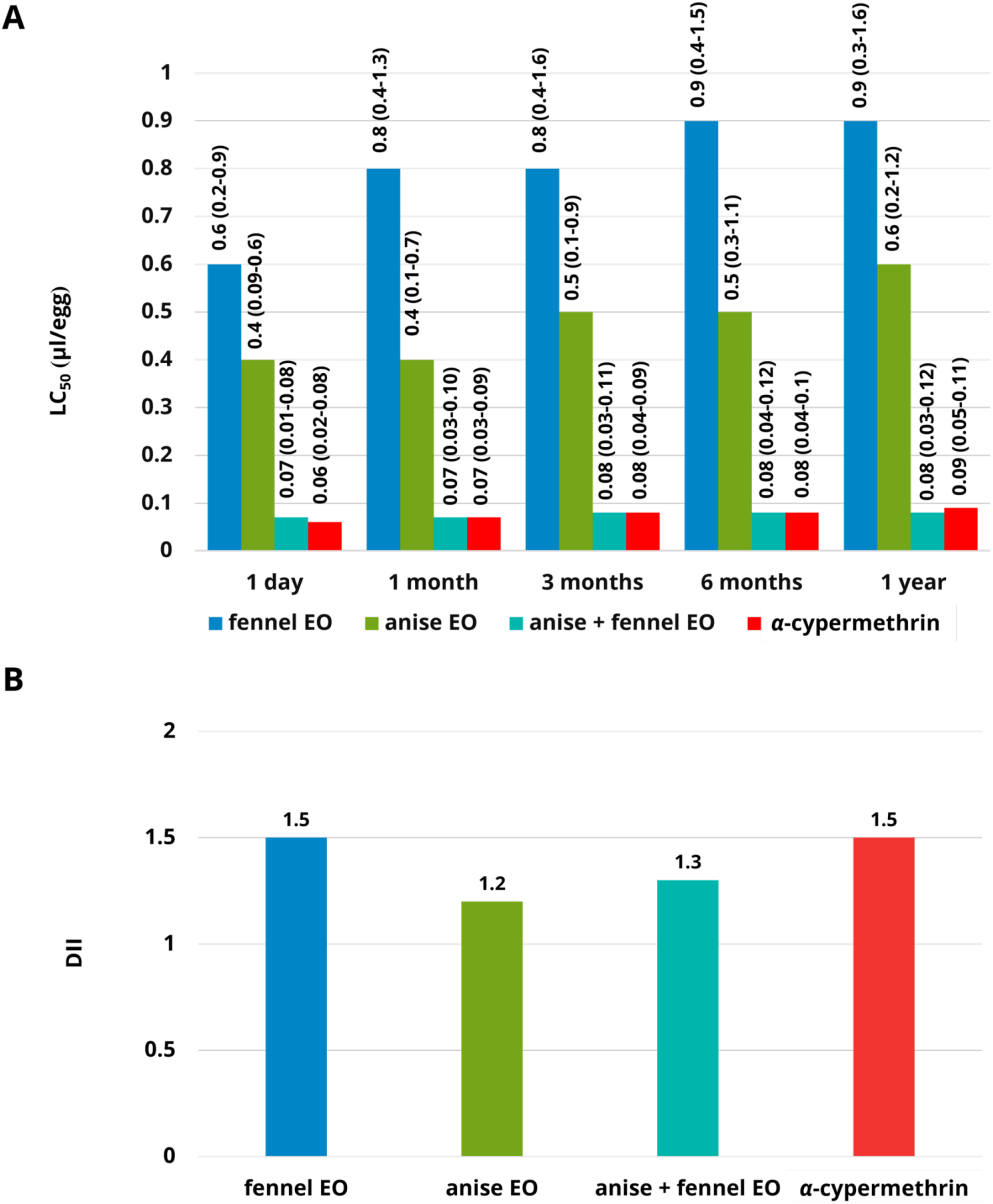
Median lethal concentration (LC_50_) **(A)** and decreasing inhibition index (DII) **(B)** of treatments against housefly eggs when compared the LC_50_ of each treatment at 1 day to the LC_50_ of each treatment at 1 year. A high DII implies lower efficacy and stability than a lower DII.

**Figure 2** presents the ovicidal efficacy expressed as median lethal concentration (LC_50_) (**Figure 2A**) and the degradation/inhibition index (DII) (**Figure 2B**). Across all five storage periods, the individual essential oil (EO) treatments exhibited LC_50_ values ranging from 0.6 to 0.9 µL per egg for fennel EO and from 0.4 to 0.6 µL per egg for anise EO. In contrast, the combined EO formulation demonstrated markedly lower LC_50_ values, ranging from 0.07 to 0.08 µL per egg, while α-cypermethrin showed LC_50_ values between 0.06 and 0.09 µL per egg. A gradual decline in ovicidal activity was observed for all treatments with increasing storage duration. Over a one-year period, both individual EOs exhibited increases in DII values by approximately 1.2–1.5-fold, indicating reduced stability. Anise EO maintained relatively higher activity (DII = 1.5) compared with fennel EO (DII = 2.0). The combined EO formulation displayed superior efficacy and stability, with a DII value of 1.3, representing greater stability than both single EOs and α-cypermethrin, which showed a DII of approximately 1.5.

**Figure 3:**
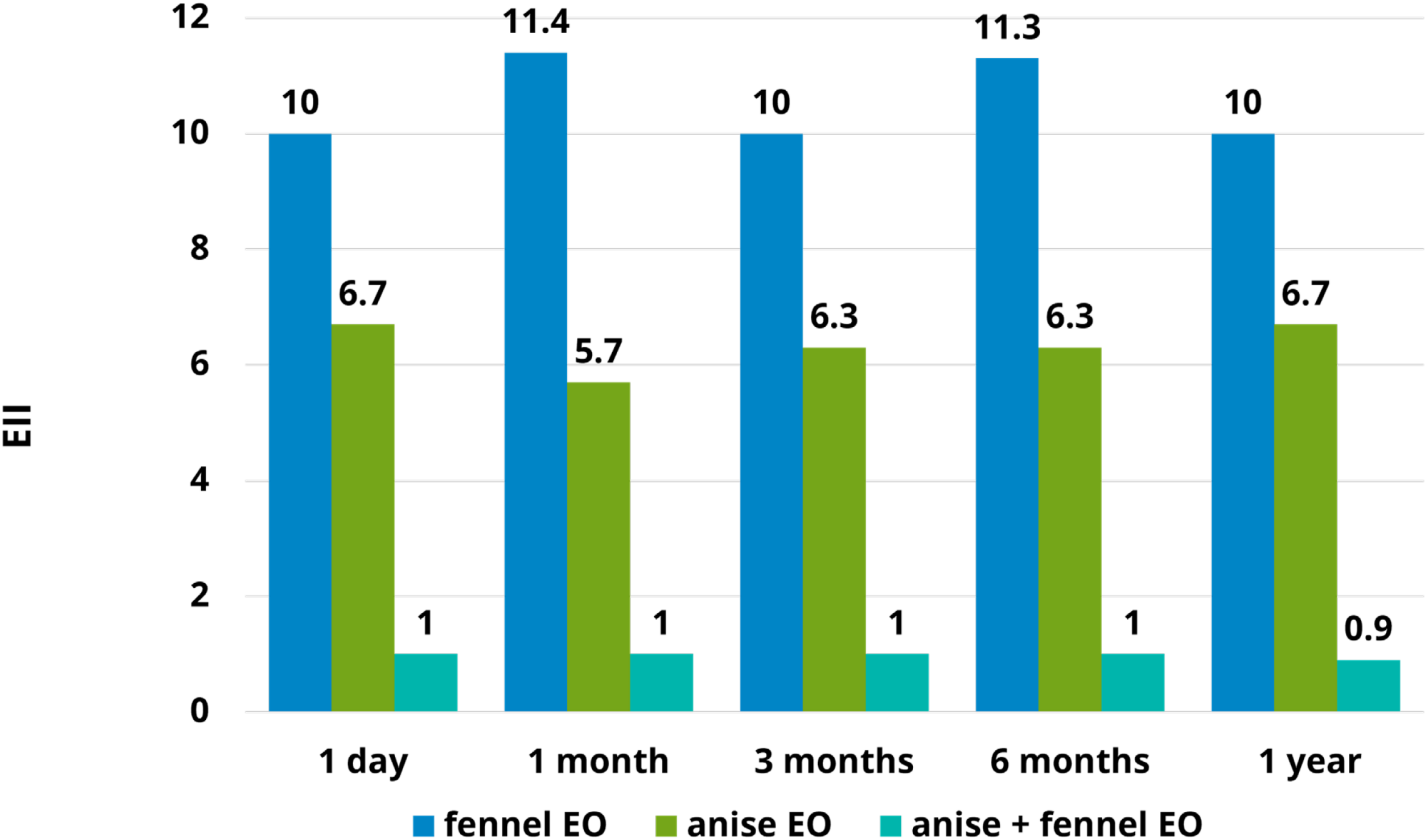
Effective inhibition index (EII) of single and combined EO formulations versus storage time from 1 day to 1 year when compared to α-cypermethrin.

**Figure 3** illustrates the ovicidal efficacy against housefly eggs expressed as the effective inhibition index (EII) relative to α-cypermethrin. Both individual essential oils (EOs) exhibited lower ovicidal activity than α-cypermethrin, with EII values ranging from 5.7 to 11.4. In contrast, the combined EO formulation demonstrated efficacy comparable to α-cypermethrin during storage periods of 1 to 6 months. Notably, after 1 year of storage, the EO combination displayed greater ovicidal activity than α-cypermethrin, with an EII value relative to cypermethrin of 0.9.

**Figure 4:**
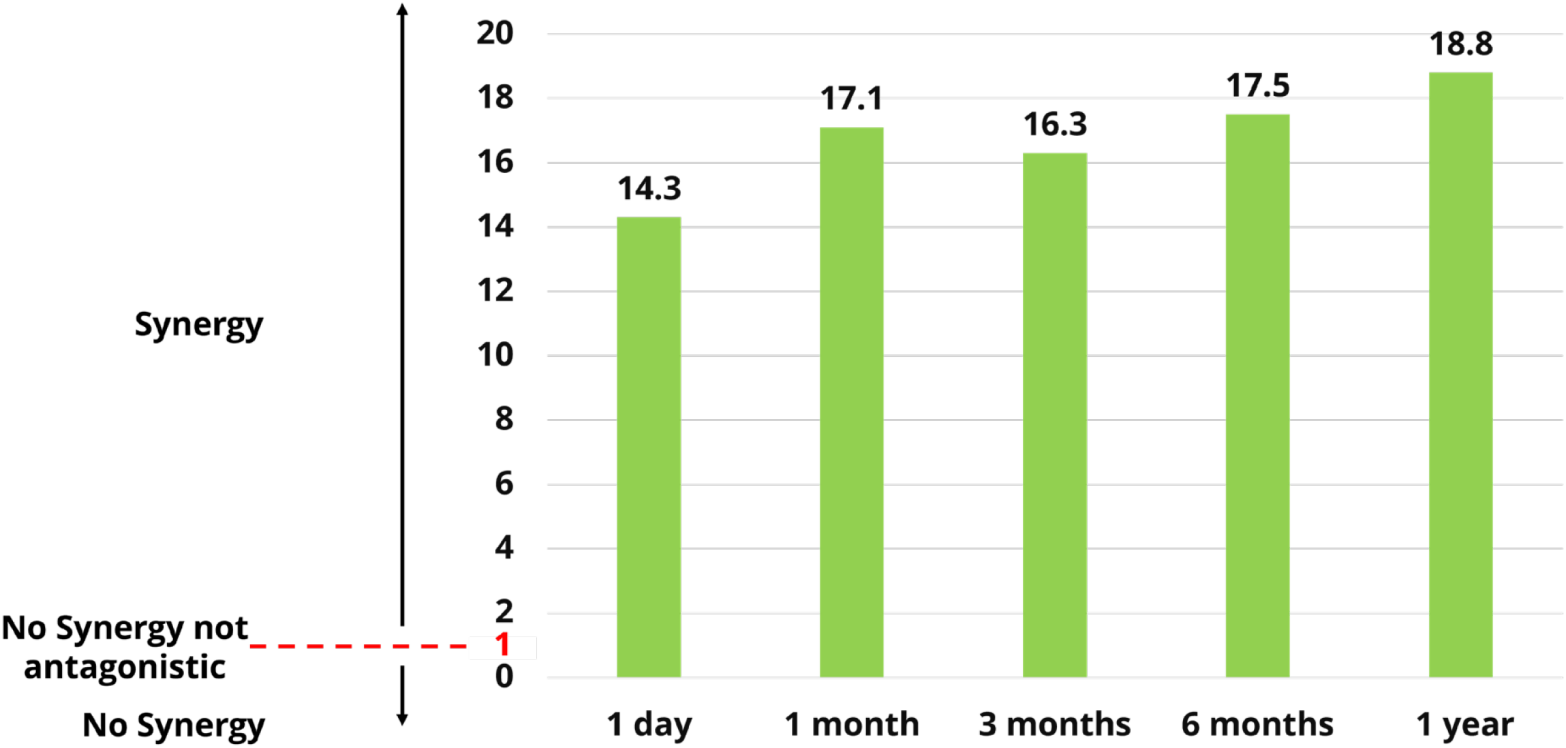
Synergistic Inhibition Index (SII) of the combined EO formulation against housefly eggs versus storage time from 1 day to 1 year when compared to single EO formulations.

Furthermore, the combined EO formulation consistently exhibited higher ovicidal efficacy against housefly eggs across all evaluated storage periods compared with single EO formulations, as indicated by synergistic inhibition index (SII) value ranging from 14.3 to 18.8. Notably, the synergistic effect remained stable over time and was sustained at a high level throughout the one-year storage period (**Figure 4**).

### 3.2 Morphological change after ovicidal bioassay

**Figure 5:**
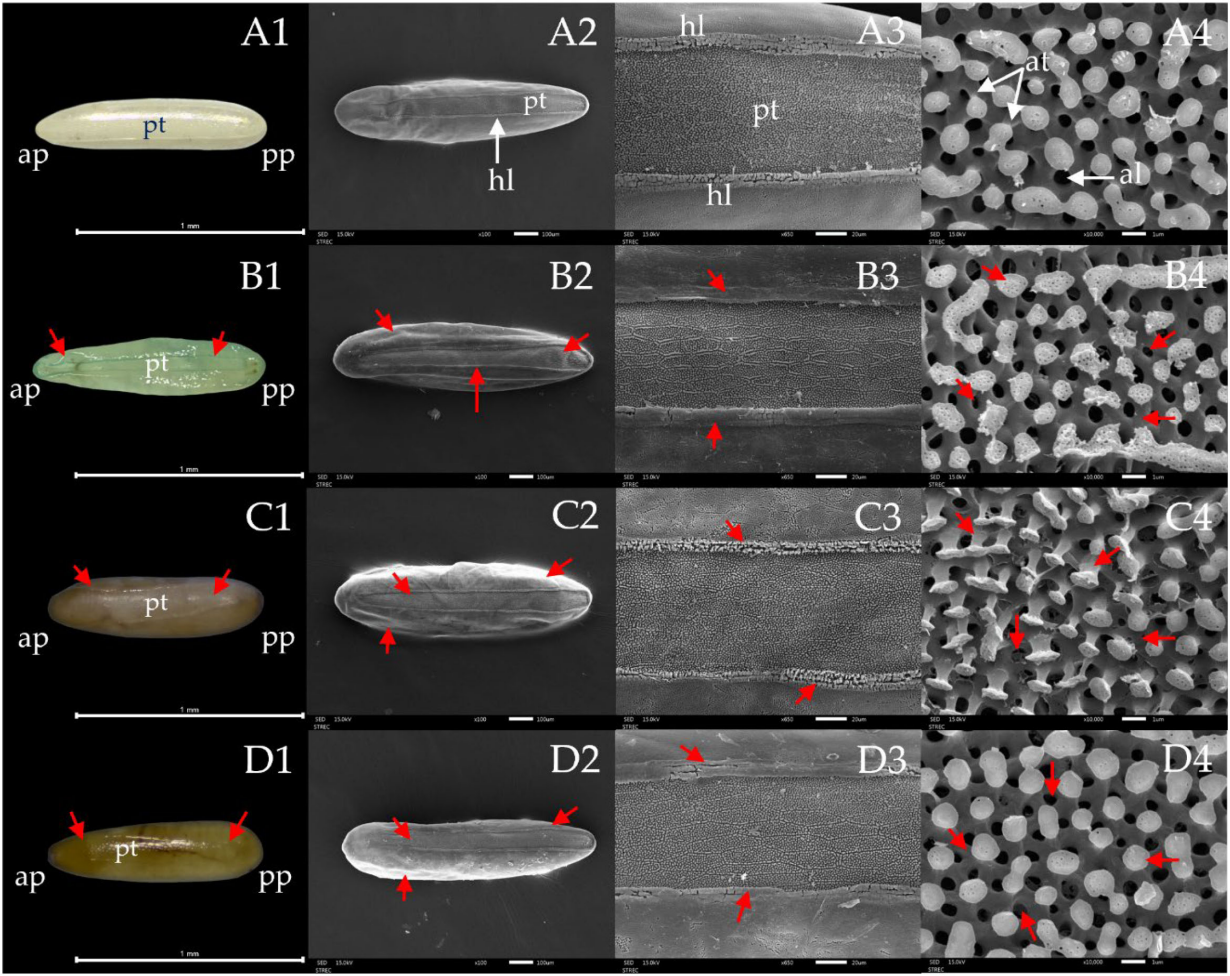
Optical and scanning electron micrographs of housefly eggs. Line **(A)** shows untreated egg with normal morphology **(A1)**, normal anterior pole (ap, left) and posterior pole (pp, right) **(A2)**, normal hatching line (hl) and normal plastrons (pt) **(A3)**, and normal islands with normal anastomosis (at) and aeropyles (al) **(A4)** (white arrow). Lines **(B–D)** show damages caused by EOs, with abnormal shapes, swollen and damaged hatching lines and plastrons, and dead embryo inside the egg after the eggs were treated with fennel EO **(B1–B3)**, shriveled anastomosis **(B4)**, anise EO **(C1–C3)**, deformed anastomosis **(C4)**, anise + fennel combination **(D1–D3)**, and swollen anastomosis **(D4)** (red arrow).

After 48 h of exposure to the individual essential oils (EOs) and their 1:1 combined EO formulation, both external and internal morphological abnormalities in housefly eggs were documented using optical microscopy and scanning electron microscopy (SEM) (**Figures 5A–D**). Eggs treated with fennel EO (B1–B4), anise EO (C1–C4), and the combined anise + fennel EO formulation (D1–D4) exhibited pronounced structural deformities, including visible alterations in egg shape, damage to the eggshell, disruption of hatching lines and plastrons, and the presence of dead embryos. In contrast, eggs in the untreated group (A1–A4) displayed intact eggshells, well-defined hatching lines and plastrons, and normally developed embryos.

### 3.3 Toxicity to earthworms

The toxicity of single EO formulations and their combined formulation at concentrations ranging from 100 to 400 µL/kg. was evaluated based on adult mortality following a 15-day exposure period (**Table 2**). None of the EO treatments exhibited toxic effects on earthworms at any of the tested concentrations. In contrast, α-cypermethrin showed pronounced toxicity, resulting in up to 100% mortality at a concentration of 200 µL/kg. Moreover, α-cypermethrin caused substantial toxicity even at lower application rate, with approximately 87% mortality observed at 100 µL/kg.

**Table 2:** Effects of different treatments on the non-target invertebrate *E. fetida* at 15 days after exposure.

Treatment	Concentration (µL/kg)	Mortality rate (%) (Mean ± SD)			
		Day 1	Day 5	Day 10	Day 15
anise EO	100	0^c^	0^c^	0^c^	0^c^
	200	0^c^	0^c^	0^c^	0^c^
	400	0^c^	0^c^	0^c^	0^c^
fennel EO	100	0^c^	0^c^	0^c^	0^c^
	200	0^c^	0^c^	0^c^	0^c^
	400	0^c^	0^c^	0^c^	0^c^
anise + fennel EO	100	0^c^	0^c^	0^c^	0^c^
	200	0^c^	0^c^	0^c^	0^c^
	400	0^c^	0^c^	0^c^	0^c^
distilled water	100	0^c^	0^c^	0^c^	0^c^
	200	0^c^	0^c^	0^c^	0^c^
	400	0^c^	0^c^	0^c^	0^c^
*α*-cypermmethrin	100	0^c^	0^c^	52 ± 1.48^b^	86 ± 1.34^b^
	200	38 ± 1.3^b^	100^a^	100^a^	100^a^
	400	48 ± 1.79^a^	100^a^	100^a^	100^a^
	ANOVA*_F_*_0.01_, D*f_total_*	**, 74	**, 74	**, 74	**, 74
	*P-*value	*P* < 0.01	*P* < 0.01	*P* < 0.01	*P* < 0.01

Values that are accompanied by different letters (a-c) indicate significant differences between them, as determined by ANOVA and Tukey’s tests. ** *p* < 0.01.

## 4 Discussion

Plant essential oils (EOs) showed promising and eco-friendly suppression of housefly due to their strong ovicidal and repellent activities, at many instances more than 90% efficacy (11, 20). The other study demonstrated that EO-based formulations exert multiple biological activities on houseflies: insecticidal, ovicidal, and oviposition deterrent (13, 14). These formulations control the pest at multiple life stages, increasing the overall control efficacy and minimizing population buildup (38). Ovicidal action is one of the most crucial modes, because egg hatching is directly related to limitation in population recruitment, the absence of emergence of successive generations reduces the pathogen transmission risk from houseflies (11–14). One of the most important advantages of using EOs for pest control was the highly synergistic EO formulation from blending oils obtained from different plant species. In the present study, EO combinations, especially that of anise and fennel showed better ovicidal and adulticidal efficacy than single EOs. This type of synergistic effect can reduce the concentration or dosage at which effective pest control is achieved, thus reducing environmental exposure and increasing safety in formulation (11). The present results are in good agreement with those previously reported by Passara et al. (11). Taken together, these findings highlight the importance of EO synergism.

The uniformity of EO synergism is also corroborated through comparable results obtained in other medico-economically important insects. For instance, a complete inhibition of egg hatching in mosquitoes (*Aedes aegypti* and *Aedes albopictus*), rapid contact toxicity towards them, and a potent anti-gravid female deterrent effect were well-documented for several EO mixtures (39, 40). Moreover, a high ovicidal effect of EO blends was also reported on eggs of American cockroach (*Periplaneta americana*) (41) as well as head lice (*P. humanus*) (15), suggesting the multi-insect potential of EO-based formulations in various insect groups. These interspecies responses are indicative that EO-based ovicides could be a broad-spectrum platform technology useful for IPM.

Although EOs show strong biological activity, EO-based products are often criticized for their volatility, storage stability, and loss of activity after long-term storage (11, 16, 42). Crucially, our study shows that these limitations can be successfully overcome using suitable combinations of  EOs. The product with the lowest DII value (DII = 1.3), showed a high ovicidal activity after one year of storage and without any significant changes in main physical properties: color, clarity, and odor. This long-term stability is a significant practical asset, and addresses directly one of the major obstacles for the commercial development of EO-based ovicides. These findings are in agreement with earlier studies reporting that anise + fennel EO had a lasting efficacy after an extended storage period (11, 39). Comparative efficacy analysis also underscored the clinical relevance of EO-combinations. According to the EII values, 1:1 anise + fennel EO showed an equivalent or a higher ovicidal efficacy than that of α-cypermethrin, a widely used synthetic insecticide. This observation is of considerable importance because of the high prevalence levels of resistance to α-cypermethrin that have been reported in housefly populations around the world, with corresponding resistance ratios varying from a few-fold to several hundred thousand under laboratory and field conditions (31, 43). In contrast, the EO blend acts via diverse biochemical and physical modes of action, which may retard resistance and provides sustainability in long-term control approaches.

Microscopic studies using scanning electron and optical microscopes gave a convincing mechanistic basis for ovicidal action of the combined EO formulation. Uniform structural damages to key egg structures, including hatching lines, plastrons, and aeropyles were observed after exposure to EO resulting in impaired gaseous exchange, suffocation of the embryo and egg non-hatching (11). *Trans*-anethole, a major component of anise and fennel EOs, was likely to be a key driver in this regard as its lipophilic nature allows it to penetrate into the pest eggshell and interfere with respiration pathways (11, 30). Similar structural and physiological impairments have been observed in EO-treated mosquitoes and louse eggs. Geranial treatment shows higher activity against mosquitoes, whereas D-limonene is more effective against lice (41, 44). Apart from ovicidal activities, some components of EOs also act on insect’s neurophysiology, e.g., inhibition of acetylcholinesterase activity and disruption of chemosensory processes, which can affect insects at different developmental stages (20, 45–48). Collectively, these results suggest that the combined EO formulation exhibits ovicidal activity through a combination of physical and biochemical modes of action, causing damages to cuticular micropyles and interfering with the eggshell respiratory structures and essential physiological pathways. Such multiple modes of action make EO-based formulations useful as botanicals for control of insect pests (28, 49).

Apart from their efficacy, the ecotoxicological safety of EO-based formulations is a matter for concern on the practical use of them as insecticides. Soil invertebrates, especially earthworms, have been considered sensitive and ecologically relevant indicators and are important components of the soil food web, playing vital roles in nutrient circulation, soil aeration, and decomposition of organic substances (50, 51). In the current study, all treatments of EOs ‐ including the synergistic anise + fennel combination ‐ were found to have low impact on earthworms, ensuring that the selectivity of EO treatments to target pest was selective. These results are consistent with those of the known studies with regard to the low toxicity of essential oils in soil on non-target organisms (9, 52–54). Unlike ANA treatments, some of the commonly used insect control chemicals are known to pose a high risk to earthworms. Cypermethrin and its isomers, such as  α -cypermethrin are highly toxic to earthworms even at low level of exposure (55–57). Most of these compounds are neurotoxins that cause an over excitation of the nervous system and affect voltage-dependent sodium channels in nerves causing prolonged excitation of neurons, impaired locomotion, short digging activity, and finally death (58, 59). Furthermore, the cypermethrin deposition has also been associated with oxidative stress, enzymatic imbalance, and cellular membrane damage that affect basic physiological roles like respiration, nutrient uptake, and immune response (60–62). At the population scale, such effects can result in reduced growth and reproductive performance which leads to population decline and ultimately the breakdown of soil structure, nutrient cycling, and depletion of overall soil fertility (56, 63, 64). Such negative effects have a serious impact on soil degradation and the long-term stability of the ecosystem (65–67). Importantly, our previous reports also showed that after prolonged storage the anise + fennel EO mixture was safe for non-target aquatic animals (e.g., guppies), which further confirms the environmental safety and potential fit to be used in eco-friendly pest management practice (11).

In conclusion, this study demonstrates that a 1:1 EO mixture of anise + fennel is a very strong alternative to currently used synthetic insecticides for control of houseflies. Their high ovicidal, repellent, and adulticidal properties along with long-term storage stability as well as the low toxicity to non-target soil organisms like earthworms strongly suggest that they have a great potential for integrated pest management programs. The adoption of this EO-based strategy could help to substantially decrease dependence on synthetic insecticides, inhibit the progression of fly resistance, and achieve a safer and more sustainable management of housefly populations and associated diseases.

## Data Availability

The original contributions presented in the study are included in the article/supplementary material. Further inquiries can be directed to the corresponding author.
